# 

**Published:** 1853-04

**Authors:** 


### CONTENTS

OF NO IV.

---

### ORIGINAL COMMUNICATIONS.

### ESSAYS AND CASES.

ART. I.—Creasote in Menorrhagia. By Dr. JOHN W. CROOKS, of Rockport, Ind. - - - - - - - 279

ART. II.—Opium in acute Inflammation. An Inaugural Dissertation, by EDWARD COOKE, M. D. Submitted to the Trustees and Medical Faculty of the University of Louisville, February, 1853. - - - - - - 281

### REVIEWS.

ART. III.—Principles of Human Physiology, with their chief applications to Psychology, Pathology, Therapeutics, Hygiene, and Forensic Medicine. By WILLIAN B. CARPENTER, M. D., F. R. S., F. G. S., Examiner in Physiology and Comparative Anatomv in the Universsty of London, Professor of Medical Jurisprudence in University College, etc. Fifth American from the fourth enlarged London edition, with three hundred and fourteen illustrations. Edited, with additions, by FRANCIS GURNEY SMITH, M. D., Professor of the Institutes of Medicine in the Medical Department of Pennsylvania College, Lecturer on Physiology in the Philadelphia Association for Medical Instruction, etc. - - - - 309

SELECTIONS FROM FOREIGN AND HOME JOURNALS.

Kentucky Surgery. - - - - - - - - - - 327
Lithotomy and Calculous Diseases. - - - - - - - 334
Self-Castration. - - - - - - - - - 340
Treatment of Spontaneous Aneurism by Rest and Absolute Diet. 342
Case of Abscess of the Brain. - - - - - - - - 343
New Effect of the Administration of Chloroform. - - 344
Calculous Diseases in China. - - - - - - - - 345
Extract from Bartlett's Discourse on Hipppocrates. - - 346
Catalepsy. - - - - - - - - - - - 349
Bite of the Rattlesnake. - - - - - - - - - 350
Analysis of Mineral Waters. - - - - - - - - 352
Congenital Occlusion of the Vagina. - - - - - 354
Yeast in Diabetes. - - - - - - - - - - 357
Munificent Gifts to Medical Institutions by M. Orfila. - 360
Extraordinary Birth. - - - - - - - - - - 362

ORIGINAL INTELLIGENCE.

Death of Professor Horner. - - - - - - - - 363
Obituary Notice of Professor Power. - - - - - 365
La Roche on Yellow Fever. - - - - - - - - 367
The Virginia Medical and Surgical Journal. - - - 368
Chloroform—Preventive of Puerperal Mania. - - - - 368
Monstrosities. - - - - - - - - - - - 369
Catalogue of the Graduates of the University of Louisville. 369
Medical Literature of Kentucky. - - - - - - 370

